# Relationship of PIEZO1 and PIEZO2 vascular expression with diabetic neuropathy

**DOI:** 10.3389/fphys.2023.1243966

**Published:** 2023-11-20

**Authors:** Yolanda Garcia-Mesa, Roberto Cabo, Mario González-Gay, Jorge García-Piqueras, Eliseo Viña, Irene Martínez, Teresa Cobo, Olivia García-Suárez

**Affiliations:** ^1^ Grupo SINPOS, Department of Cell Biology and Morphology, University of Oviedo, Oviedo, Spain; ^2^ Instituto de Investigación Sanitaria del Principado de Asturias, ISPA, Oviedo, Spain; ^3^ Sercivio de Angiología y Cirugía Vascular, Fundación Hospital de Jove, Gijón, Spain; ^4^ Servicio de Anatomía, Histología y Neurociencias, Universidad Autonoma de Madrid, Spain; ^5^ Servicio de Cardiología, Unidad de Hemodinámica y Cardiología Intervencionista, Hospital de Cabueñes, Gijón, Spain; ^6^ Servicio de Cirugía Plástica y Reparadora, Fundación Hospital de Jove, Gijón, Spain; ^7^ Departamento de Cirugía y Especialidades Médico-Quirúrgicas, Universidad de Oviedo, Oviedo, Spain; ^8^ Instituto Asturiano de Odontología S.L, Oviedo, Spain

**Keywords:** diabetic distal symmetrical polyneuropathy, Piezo, painful and painless, DPN, skin, blood vessels

## Abstract

**Introduction:** Diabetic distal symmetric polyneuropathy (DDSP) is the most prevalent form of diabetic peripheral neuropathy, and 25% of patients develop pain in their toes. DDSP is associated with increased cutaneous microvessel density (MVD), reduced skin blood flow, endothelial dysfunction, and impaired fluid filtration with vasodilation. The Piezo family of mechanosensitive channels is known to be involved in the control of vascular caliber by converting mechanical force into intracellular signals. Furthermore, Piezo2 is particularly involved in peripheral pain mechanisms of DDSP patients. To date, very little is known about the number, structure, and PIEZO expression in cutaneous blood vessels (BVs) of individuals with DDSP and their relation with pain and time span of diabetes.

**Methods and results:** We studied microvessels using endothelial markers (CD34 and CD31) and smooth cell marker (α-SMA) by indirect immunohistochemical assay in sections of the glabrous skin of the toes from patients and controls. MVD was assessed through CD34 and CD31 immunoreaction. MVD determined by CD34 is higher in short-term DDSP patients (less than 15 years of evolution), regardless of pain. However, long-term DDSP patients only had increased BV density in the painful group for CD31. BVs of patients with DDSP showed structural disorganization and loss of shape. The BVs affected by painful DDSP underwent the most dramatic structural changes, showing rupture, leakage, and abundance of material that occluded the BV lumen. Moreover, BVs of DDSP patients displayed a Piezo1 slight immunoreaction, whereas painful DDSP patients showed an increase in Piezo2 immunoreaction.

**Discussion:** These results suggest that alterations in the number, structure, and immunohistochemical profile of specific BVs can explain the vascular impairment associated with painful DDSP, as well as the temporal span of diabetes. Finally, this study points out a possible correlation between increased vascular Piezo2 immunostaining and pain and decreased vascular Piezo1 immunostaining and the development of vasodilation deficiency.

## 1 Introduction

Diabetic peripheral neuropathy (DPN) is one of the most common long-term complications of diabetes mellitus (DM) (Pop-Busui et al., 2017). A total of 25% of these patients develop pain in their toes (Bierhaus and Naworth, 2004; Boulton et al., 2005), but the mechanism behind painful and painless DPN remains unclear. The Toronto Expert group has defined DPN as a symmetrical, length-dependent sensorimotor polyneuropathy attributable to metabolic and microvessel alterations as a result of chronic hyperglycemia exposure and cardiovascular risk covariates (Tesfaye et al., 2010). Diabetic patients with DPN have altered blood flow patterns in the lower limbs (Archer et al., 1984; Tomešová et al., 2013), impairment of cutaneous endothelium-related vasodilatation, and c-fiber-mediated vasoconstriction (Quattrini et al., 2007). Beyond metabolically triggered microvascular changes induced by DM, the presence of neuropathic abnormalities is the main factor associated with microvascular abnormalities (Schmiedel et al., 2008; Tomešová et al., 2013; Stirban, 2014). Patients with DPN show only partial improvement in microvascular function after lower extremity revascularization, highlighting the pathogenic contribution of DPN to microvascular dysfunction (Arora et al., 2002). The presence of DPN is associated with increased microvessel density (MVD) in the skin, reduced cutaneous blood flow, endothelial dysfunction, impairment in fluid filtration, and vasodilatation (Stirban, 2014; Adamska et al., 2019). Several studies have shown that regulation of the peripheral blood flow is altered in patients with painful DPN, compared with painless DPN (Archer et al., 1984; Quattrini et al., 2007; Doupis et al., 2008). Moreover, endothelial dysfunction and the increased vascular density were associated with the severity of pain in patients with diabetic neuropathy (Quattrini et al., 2007; Shillo et al., 2019; 2021).

Mechanotransduction is the process through which mechanical forces are translated to electrical inputs. The physiological response is very important for local blood flow control and regulation of vascular tone (Ge et al., 2015; Rode et al., 2017; Beech and Kalli, 2019; Arishe et al., 2020). In the last decade, mechanosensitive channels Piezo1 and Piezo2 have been implicated in the control of vascular function, converting mechanical forces (shear stress and stretch) into intracellular signals. Both are present in the vascular system on either the endothelial cell (EC) membrane and the vascular smooth muscle (VCM) (Ranade et al., 2014; Retailleau et al., 2015; Wang et al., 2016; Yang et al., 2016; Li et al., 2017; Martinac and Cox, 2017; Rode et al., 2017; Otto et al., 2020; Wang et al., 2022).

The mechanosensitive channel Piezo1 is required for adequate formation of blood vessels (BVs) (Cahalan et al., 2015) and regulation of vascular tone and blood pressure (Wang et al., 2016; Rode et al., 2017; Wu et al., 2017). Piezo1 appears to function in endothelial cell types under both static and shear stress conditions. In fact, mice with endothelium-specific disruption of Piezo1 also exhibited abnormal vessel formation and perturbed endothelial cell organization with alignment in the direction of the flow (Li J et al., 2014; Ranade et al., 2014; Nourse and Pathak, 2017; Fang et al., 2021). Recent studies have shown the considerable importance of Piezo1 as a mechanical sensor responsible for endothelial barrier disruption in the lungs (Friedrich et al., 2019), and shear stress-induced sensitization of Piezo1 increases the membrane density of Piezo1 channels in endothelial cell cultures (Lai et al., 2021).

The mechanosensitive channel Piezo2 is present in the endothelial cell lining the lumen of BVs (Ferrari et al., 2015; Martinac and Cox, 2017). Piezo2 is known to regulate endothelial cell proliferation, migration, and tube formation in tumor vasculature (Yang et al., 2016), and the experimental knockdown of Piezo2 shows decreased angiogenesis, vascular hyperpermeability, and vascular leakage in tumor endothelial cells (Yang et al., 2016). In addition, the endothelial Piezo2 channel plays a role in peripheral pain mechanisms of painful peripheral neuropathy produced by cancer chemotherapy (Joseph et al., 2013; Ferrari et al., 2015).

Overall, both Piezo channels play a role in the miscellaneous aspects of the microvascular dysfunction and could be, therefore, a possible clinical target for the treatment of diabetic complications such as pain or diabetic foot.

To date, despite considerable dedicated research, a full understanding of neuropathic pain mechanisms remains difficult to achieve. In fact, there are few distinct differences in the neurological examination between painless and painful DPN, and it has been suggested that an inappropriate skin microcirculation may play a role in the pathogenesis of pain in diabetic neuropathy (Quattrini et al., 2007; Shillo et al., 2021; Wang et al., 2022), but no in-depth study has been carried out. Therefore, the identification of BV distinctive aspects that could characterize painful or painless DDPN in short- and long-term diabetes is the purpose of this study. Here, we studied the glabrous skin of the toes of patients clinically and analytically diagnosed with DM with painless or painful diabetic neuropathy. We used immunohistochemistry to detect CD31, CD34, and alpha-smooth muscle actin (α-SMA) markers to analyze the MVD, microvessel localization within the dermis, arrangement of BV components (EC and VCM or pericytes), and expression of mechanosensitive Piezo channels in cutaneous BVs of subjects with painless and painful DPN. Finally, we analyzed if differences between the groups could be associated with pain and diabetes duration.

## 2 Materials and methods

### 2.1 Patients

The control samples were obtained from subjects of both genders, free of neurologic disease, who suffered accidental toe amputation (*n* = 10) and were collected within 6 h after incidental toe amputation at the Service of Plastic Surgery of the Hospital Universitario Central de Asturias, Oviedo, Principality of Asturias, Spain. Patients clinically and analytically diagnosed with DM with painless (n = 10) or with painful diabetic neuropathy (*n* = 10), who were subjected to toe amputation due to ischemic complications of DM, were also studied. The age range was 48–84 years. Each group with diabetic distal symmetric polyneuropathy (DDSP) was divided into two subgroups depending on the time of evolution from the diagnosis of DM: short evolution (ST) if less than 15 years and long evolution (LT) if more than 15 years. The skin samples from diabetic patients were collected within 3 h after amputation and were obtained at the Service of Vascular Surgery, Fundación Hospital Jove of Gijón, Principality of Asturias, Spain. These materials were all obtained in compliance with Spanish law (RD 1301/2006; Ley 14/2007; DR 1716/2011; Orden ECC 1414/2013) and according to the guidelines of the Helsinki Declaration II. The study was approved by the Ethical Committee for Biomedical Research of the Principality of Asturias, Spain (Cod. CElm, PAst: Proyecto 266/18).

The subjects included in this study were used previously in our published manuscript about changes in cutaneous sensory corpuscles associated with painful and painless DPN (García-Mesa et al., 2021).

### 2.2 Materials and treatment of the tissues

Skin samples (*n* = 30) were obtained from the plantar aspect of the distal phalanx of amputated toes. Tissues were fixed in 4% formaldehyde diluted in 0.1 M phosphate buffer saline (pH 7.4) for 24 h, dehydrated, and routinely embedded in paraffin.

### 2.3 Histology and immunohistochemistry

#### 2.3.1 Single immunohistochemistry

Deparaffinized and rehydrated 7 µm sections were processed for indirect detection of antibodies (Table 1), using the EnVision antibody complex detection kit (Dako, Copenhagen, Denmark), following the supplier’s instructions. In brief, the endogenous peroxidase activity was inhibited (3% H2O2) for 15 min, and 10% bovine serum albumin (BSA) was used to block non-specific binding for 20 min. The sections were incubated overnight at 4°C with the primary antibody. After that, the sections were incubated with the anti-rabbit and anti-mouse EnVision system-labeled polymer (Dako-Cytomation) for 30 min and washed in a buffer solution. Then, the slides were washed with buffer solution, and the immunoreaction was visualized with diaminobenzidine as a chromogen. Finally, the sections were washed, dehydrated, and mounted with Entellan^®^ (Merck, Dramstadt, Germany). The sections were counterstained with Mayer’s hematoxylin to ascertain structural details.

**TABLE 1:** Primary antibodies used in the study.

Blood vessel marker			
Antigen	Origin	Dilution	Supplier
CD31	Mouse	Pre-diluted	Leica Biosystems, Spain
CD34	Mouse	Pre-diluted	Leica Biosystems, Spain
α-SMA	Mouse	Pre-diluted	Leica Biosystems, Spain

Ion channels						
Antigen	Origin	Dilution	Supplier	Stock	Epitope	Homology
PIEZO1 (LS-B156/12883)^a^	Rabbit	1:200	Invitrogen	In stock	UniProt Q92508	See https://www.uniprot.org/uniref/UniRef90_Q92508
			PA5-72974			
			Lote: VL3144473A			
PIEZO2 (63895)^b^	Rabbit	1:500	Invitrogen	In stock	UniProtKB Q9H5I5	See https://www.orthodb.org/?ncbi=63895
			PA5-56894			
			Lote: A96829			

^a^ PIEZO1: Synthetic peptide C-EDLKPQHRRHISIR.

^b^ PIEZO2: Synthetic peptide VFGFWAFGKHSAAADITSSLSEDQVPGPFLVMVLIKFGTMVVDRALYLRK.

#### 2.3.2 Double immunofluorescence

The sections were processed for simultaneous detection of PIEZO2 (targeted against the FEDEN-KAAVRIMAGDNVEICMNLDAASFSQHNP amino acid sequence) with specific markers CD34 and alpha-SMA for the endothelium and muscular layer of BVs, respectively (DeLisser et al., 1997; Matsumura et al., 1997; Zhou et al., 2022) (Table 1). Non-specific binding was reduced using a solution of 25% calf bovine serum in a Tris buffer solution (TBS) for 30 min. The tissues were incubated overnight at 4°C in a humid chamber with a 1:1 v/v mixture of the polyclonal antibody against Piezo2 with a monoclonal antibody against CD34 and alpha-SMA. After that, the sections were washed with TBS and incubated for 1 h with CFL488-conjugated bovine anti-rabbit IgG (sc-362260, Santa Cruz Biotechnology), diluted 1:200 in TBS, then rinsed again, and incubated for another hour with the CyTM3-conjugated donkey anti-mouse antibody (Jackson-ImmunoResearch, Baltimore, MD, United States) diluted 1:100 in TBS. Both steps were performed at room temperature in a dark humid chamber. Finally, the sections were washed, and the cell nuclei were stained with DAPI (10 ng/mL).

The Leica DMR-XA automatic fluorescence microscope (Microscopía fotónica y Proceso de imagen, Servicios científico-técnicos, Universidad de Oviedo) coupled with Leica Confocal Software, version 2.5 (Leica Microsystems, Heidelberg GmbH, Germany), was used to detect triple fluorescence, and the images captured were processed using the software ImageJ version 1.43 g Master Biophotonics Facility, Mac Master University Ontario (www.macbiophotonics.ca).

For control purposes, representative sections were processed in the same way as described previously, using non-immune rabbit or mouse sera instead of the primary antibodies or omitting the primary antibodies in incubation. Furthermore, in some cases, additional controls were carried out using specifically preabsorbed antisera. Under these conditions, no positive immunostaining was observed (data not shown). Positive controls for Piezo1 and Piezo2 were performed previously to confirm their specificity (Supplementary Material S1).

#### 2.3.3 Semiquantitative study

Semiquantitative analyses were performed to determine the MVD using the “hot spot technique” (Adamska et al., 2019). The sections were scanned by using an SCN400F scanner (Leica, Leica Biosystems™), and the scans were computerized using SlidePath Gateway LAN software (Leica, Leica Biosystems™). The number of BVs was calculated as follows: in each section, 10 areas with the highest number of BVs (hot spot) were identified under a small magnification (×10) and counted under a magnification of ×40 in a selected area by two independent observers (YG-M and O. G-S). We examined 10 hot spots in each tissue section (five from the papillary dermis and five from the reticular dermis). The arithmetic mean of the five “hot spots” in each area was calculated for the microvessel number and subsequently calculated to 1 mm^2^. We quantified 10 hot spots in five sections separated by 50 µm per sample in the glabrous toe skin from all groups: healthy individuals (*n* = 10), NP-DDSP-ST (*n* = 5), NP-DDSP-LT (*n* = 5), NP-DDSP-ST (*n* = 5), and P-DDSP-LT (*n* = 5). Data are expressed as mean ± SD/mm^2^. This procedure was applied separately for CD34 and CD31. We used CD34 (endothelium and pericytes) and CD31 (endothelium) as markers for morphological and functional endothelial integrity (Yao et al., 2007).

#### 2.3.4 Clinical measurement

Clinical data management was divided into five parts though a checklist:

1 Clinical history: Age, sex, variant of peripheral neuropathy, HbA1c, proinflammatory and inflammatory factors (c-reactive protein and erythrocyte sedimentation rate), alterations in the blood clotting test, and nervous conduction studies.
2 Physical examination: Maintained local sensibility, pedal pulse assessment, skin alterations or deformities, and allodynia/hyperalgesia/paresthesia/anesthesia. Sensitivity was focused on in the clinical examination, as long as anatomical structures and biochemical channels in study are responsible of this sensation.
3 Vascular examination: Ankle–brachial index and Doppler valued the alteration of the microvascular distal system.
4 DM evolution: The evolution from the diagnosis of DM was measured in years.
5 DN4 test to estimate neuropathic pain: Previous studies describe how alterations on these sensory structures may produce extreme effects in the form of a total anesthesia in the studied region or even excessive painful responses under normally painless stimuli.

## 3 Results

### 3.1 Semiquantitative analyses of cutaneous blood vessels: the association between density, neuropathy, and diabetes duration

In this work, subjects under study were classified into five groups: healthy individuals (*n* = 10), painless ST-DDSP (*n* = 5), painful ST-DDSP (*n* = 5), painless LT-DDSP (*n* = 5), and painful LT-DDSP (*n* = 5). The limit to consider long-term DDSP was 15 or more years of illness arbitrarily.

**FIGURE 1:**
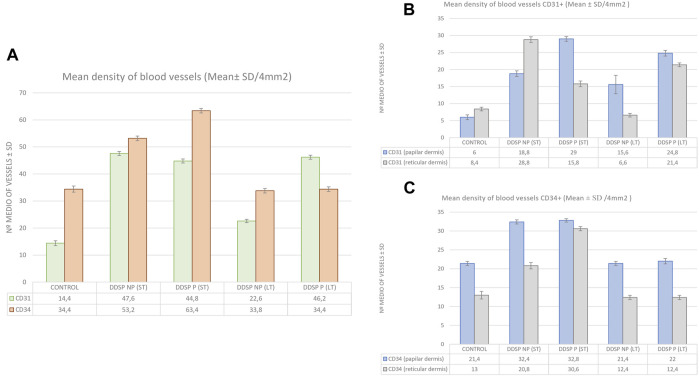
Bar graph showing the microdensity blood vessels/4 mm^2^ defined by CD31 and CD34 in the glabrous toes skin of healthy individuals (*n* = 10), DDSP-NP ST (*n* = 5) and LT (*n* = 5), and DDSP-P ST (*n* = 5) and LT (*n* = 5). Comparison of the number of blood vessels positive for CD31 (green) and CD34 (orange) in the dermis **(A)**; comparison of CD31-positive blood vessels in the papillary (blue) vs. reticular (gray) dermis **(B)**; and comparison of CD34-positive blood vessels in the papillary (blue) vs. reticular (gray) dermis **(C)**. ST, short term; LT, long term. I—I is SD.

We calculated the median MVD by CD34 and CD31 BVs per 4 mm^2^ of the dermal skin; moreover, we quantified both layers of the skin dermis: the papillary and reticular dermis (Figures 1B, C).

The mean determined by the CD31 antibody immunoreaction was higher in all groups affected by diabetic neuropathy with respect to healthy controls (Table 2; Figure 1A). The comparison of MVD determined by CD31 between the papillary and reticular dermis in each group showed that the MVD was higher in all the groups, tripling the number of healthy control BVs in the papillary dermis, whereas in the reticular dermis, patients with DDPN did not show such an evident increase. In fact, the DDSP NP (LT) group showed a lower MVD (6.6) *versus* healthy individuals (8.4). CD31 antibody indicated a higher increase of BV in the papillary dermis than the reticular dermis (Table 2; Figures 1B, C). The painful DDSP group showed a higher index with respect to healthy individuals (Figures 1B).

**TABLE 2:** Comparison of groups of patients with DDPN and healthy individuals.

Variables	Healthy individuals (*n* = 10)	DDSP-NP (ST) (*n* = 5)	DDSP-P (ST)	DDSP-NP (LT)	DDSP-P (LT)
			(*n* = 5)	(*n* = 5)	(*n* = 5)
MVD CD31^+^ vessels/1 mm^2^	M: 14, 6 (14–16)	M: 48, 8 (48–50)	M: 22,8 (22–24)	M: 23 (22–24)	M: 45, 2 (44–46)
Dermis	SD: 0, 894	SD: 0, 748	SD: 0, 748	SD: 0, 632	SD: 0, 748
MVD CD34^+^ vessels/1 mm^2^	M: 34, 4 (34–36)	M: 64, 2 (63–65)	M: 33, 8 (33–35)	M: 33, 8(33–35)	M: 35, 2 (34–36)
Dermis	SD: 1, 140	SD: 0, 836	SD: 0, 836	SD: 0, 836	SD: 0, 836
MVD CD31^+^ vessels/1 mm^2^	M: 6 (5–7)	M: 18, 8 (18–20)	M: 29 (28–30)	M: 15, 6 (13–20)	M: 24, 8 (24–26)
Papillary dermis	SD: 0, 707	SD: 0, 836	SD: 0, 836	SD: 2, 701	SD: 0, 836
MVD CD31^+^ vessels/1 mm^2^	M: 8, 4 (8–9)	M: 28, 8 (28–30)	M: 15, 8 (15–17)	M: 6, 6 (6–7)	M: 21, 4 (21–22)
Reticular dermis	SD: 0, 547	SD: 0, 836	SD: 15, 8	SD: 0, 547	SD: 0, 547
MVD CD34^+^ vessels/1 mm^2^	M: 21, 4 (21–22)	M: 32, 4 (32–33)	M: 32, 4 (32–33)	M: 21, 4 (21–22)	M: 22 (21–23)
Papillary dermis	SD: 0, 547	SD: 0, 547	SD: 0, 447	SD: 0, 547	SD: 0, 707
MVD CD34^+^ vessels/1 mm^2^	M: 13 (12–14)	M: 20, 8(20–22)	M: 30, 6 (30–31)	M: 12, 4 (12-13)	M: 12, 4 (12–13)
Reticular dermis	SD: 1	SD: 0, 836	SD: 0, 547	SD: 0, 547	SD: 0, 547

MVD, microvessel density; CD, cluster of differentiation.

Data are presented as median (M).

SD, standard deviation.

The median density of microvessels determined by the CD34 immunoreaction was higher in patients with short-term DDSP NP (53.2 and 63.4), whereas those with long-term DDSP (33.8 and 35.2) had a similar number of vessels compared to the control group (34.4) (Table 2; Figure 1A). Regarding the distribution of vessels through the different dermal layers, all the groups presented a similar pattern, with MVD being higher in the papillary dermis than that in the reticular dermis (Figures 1C). It must be mentioned that the highest MVD appeared in ST-DDSP patients in both the papillary dermis (32.4 and 32.8) and the reticular dermis (20.8 and 30.6) with respect to LT-DDSP (21.4 and 22 in the papillary dermis; 12.4 in the reticular dermis for both groups).

We further analyzed the relationship between BV density (CD34 and CD31 positive) and the presence or absence of pain. When we related painful *versus* painless DDSP, we observed a greater MVD for ST-DDPN with CD34 and for LT-DDSP with CD31 (Figure 1A). On the other hand, if the painless DDSP groups are compared with each other, a notable difference emerged, i.e., there is a decrease in the number of vessels associated with a long evolution of diabetes, for both antibodies CD31 and CD34 with respect to painful DDSP, and MVD remained the same for CD31 but decreased for CD34.

Finally, if the MVD from CD31-positive BVs is compared against CD34-positive BVs, we observed that CD34 is adequate to assess the number of vessels and pain in ST-DDSP while CD31 is better for LT-DDSP (Figure 1A).

### 3.2 Cutaneous blood vessel morphology in painful and painless DDSP subjects

**FIGURE 2:**
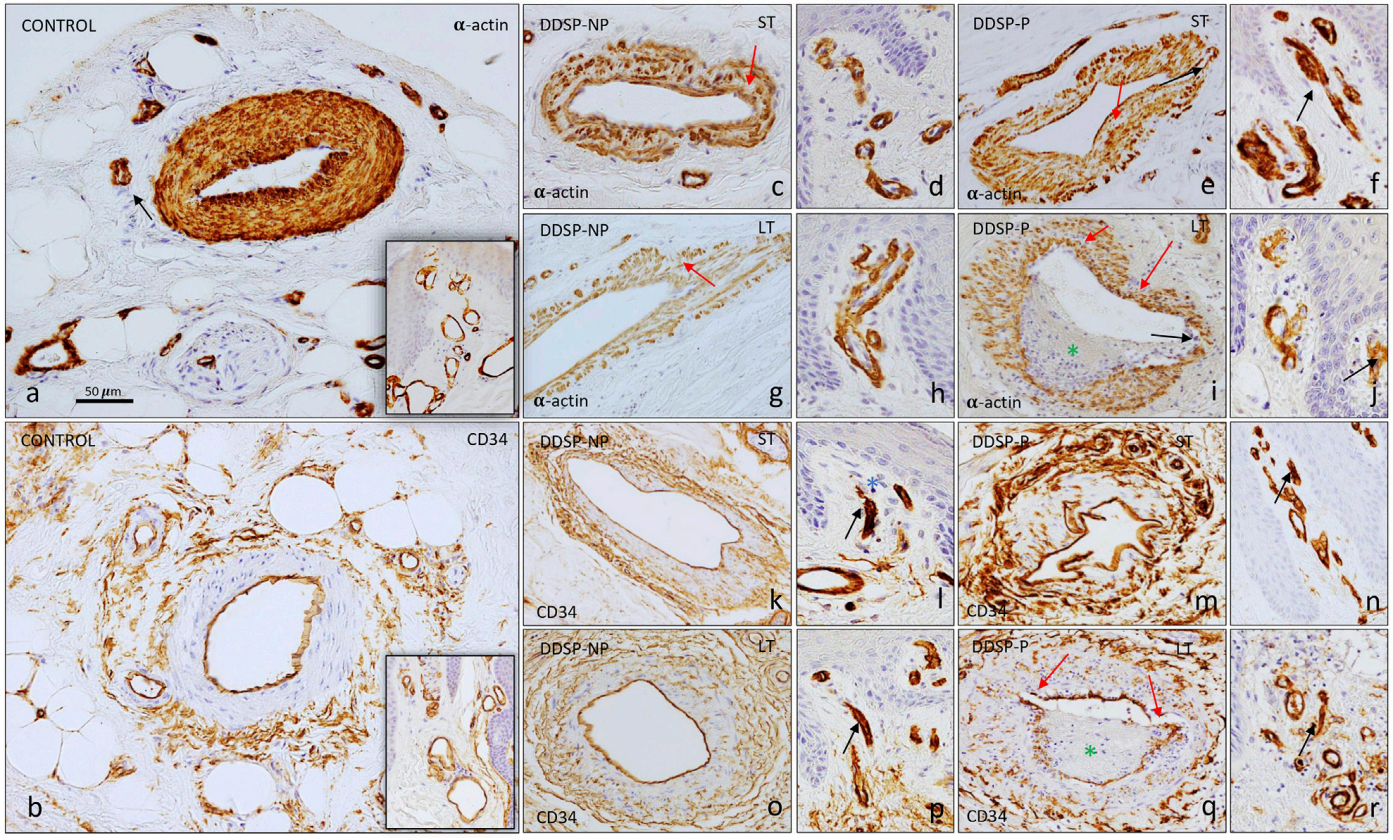
Immunostaining for α-SMA and CD34 in the glabrous toes skin. α-SMA antibody identifies the muscular layer in arteries and venules **(A, C–J)**. Blood vessels of neuropathy patients showed, in some cases, a lack of lumen [black arrow in **(F, J)**] in the papillary dermis. In the reticular dermis, the blood vessels of healthy controls present a constant muscle layer and with intense immunoreactivity **(A)**, whereas patients with diabetic neuropathy present an irregular immunostaining without a continuous α-SMA immunopositive muscle layer [see red arrows **(C, E, G)]** and the muscular layer thickness decreased [red arrow in **(I)**], even blood vessel wall was broken [black arrow in **(I)**]. CD34 immunostaining marks endothelium and single smooth muscle cells. In healthy individuals **(B)** and in patients with DDSP NP **(K, L, O, P),** a continuous and regular endothelium is observed, while patients with DDSP-P present interruptions in the course of the endothelium [red arrow in **(Q)**]. Black arrows indicate blood vessels in the papillary dermis that present a **(M,N,Q,R)** closed lumen in DPN patients [see **(L, N P, R)**]. The green asterisks indicate accumulations of debris in the lumen of the vessels **(I, Q)**.

**FIGURE 3:**
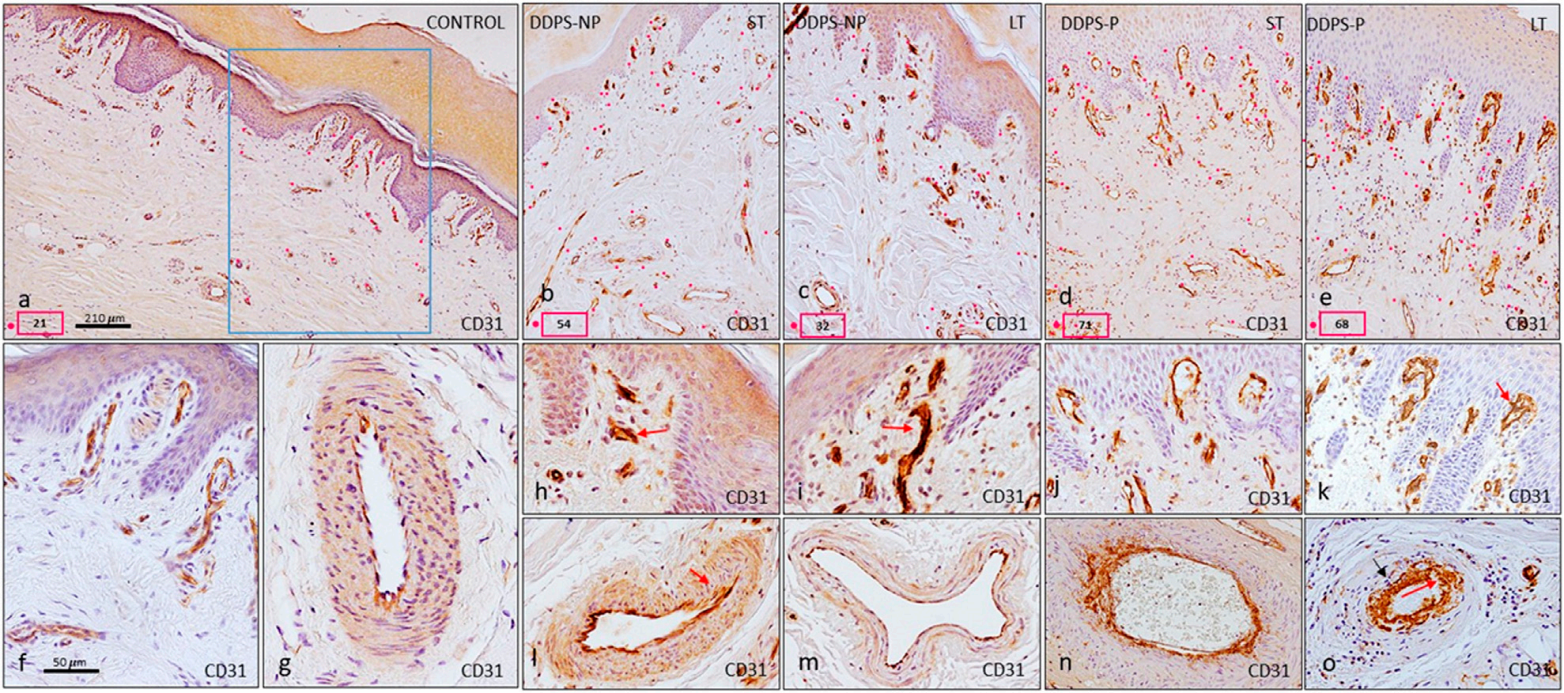
Number of microvessels in the skin of healthy adults **(A)** and with diabetes neuropathy **(B–E)**. Sections are stained by immunohistochemistry to show the number of blood vessels defined by a CD31, an endothelium marker **(A–E)**. Blood vessels are identified with a red spot in the pictures **(A–E)**. The number of blood vessels is shown in a small red box in the same images. A higher number of blood vessels are noted in patients with neuropathy, especially in DDPS-P **(D,E)**. In the papillary dermis of patients with neuropathy, **(F,H,I,J,K)** a reduced capillary luminal area [red arrow **(H,L,K)**] was observed. With respect to the reticular dermis, the immunoreaction with CD31 allows observing a continuous and regular endothelium in the healthy individuals **(F,G)**, while the patients with DDPS **(L,M,N,O)** present interruptions in the endothelium in some cases [see red arrows **(L,O)**] or multiple layers [black arrow in **(N, O)**].

BVs were analyzed in dermal layers (papillary and reticular dermis) according to immunopositive staining by using antibodies against α-SMA to detect α-smooth muscle cells and pericytes, CD34 to identify ECs and individual smooth muscle cells, and CD31 for ECs. All of them were found in both dermal layers in all experimental groups (Figures 2, 3).

#### 3.2.1 α-SMA immunoreactivity in blood vessels

Depending on the type of BV, pericytes or smooth muscle cells were tightly associated with the underlying endothelial cells in the normal skin (Figures 2A). Both types of cells can be immunolabeled with the α-SMA antibody. In the normal skin, arterioles were completely covered by circumferentially arranged α-SMA-positive cells displaying a regular morphology (Figures 2A). Venules were also completely covered by α-SMA-positive cells but had a more irregular shape with missed packing than smooth muscle cells from arterioles (Figure 2A, small square).

BVs in DDSP patients expressed α-SMA in pericytes and smooth muscle cells, which revealed that almost all BVs did not have normal morphological features. Thus, in arterioles, the typical transversal rounded shape of BVs and characteristic packing of α-SMA-positive cells were absent, leaving spaces between them (Figures 2C, E, G, I; red arrow). The most extreme changes were seen in painful DDSP due to completely irregular BVs with cellular processes projecting out of the vessel wall (Figure 2E; black arrow), which showed, in the case of long-term DDSP, a pronounced discontinuity of α-SMA-positive cells around BVs. Such vessels lost normal smooth muscle circular arrangement and presented areas of vascular wall where smooth cells almost completely disappeared and the vessel wall was about to break or had already broken (black arrow in Figures 1E, I), along with material accumulations inside (Figure 1I; asterisk in green). In the vessels located in the papillary dermis, the immunoreaction was maintained in all the study groups (Figures 1D, F, H, J), although more closed vessels were observed in DDSP-P (black arrow in Figures 1F, I).

#### 3.2.2 CD34 immunoreactivity in blood vessels

The CD34 antigen is considered a pan-immunomarker for endothelial cells as well as individual smooth muscle cells present in arterioles. The positive immunoreaction in control samples was found in the endothelial cells, which defined the perimeter of the BV lumen, forming a continuous barrier (Figure 2B). In individuals with DDSP, the typical transversal rounded shape of arterioles was absent, especially in the painful group (Figures 2K, M, O, Q). The greatest involvement was found in the group with painful long-term DDSP, presenting loss of endothelial continuity or endothelial layer rupture (red arrow in Figure 2Q) with material accumulation (green asterisk). Regarding the vessels located in the papillary dermis, all the groups studied expressed immunoreaction for CD34 in the endothelium (Figures 2L, N, P, R), as described previously in the case of α-SMA immunomarker, displaying a decrease in the vessel lumen diameter and being absent in some cases (black arrow in Figures 2L, N, P, R).

#### 3.2.3 CD31 immunoreactivity in blood vessels

In a high-magnification view of the dermis, a large number of vessels were present in the DDSP group, regardless of pain manifestation or diabetes duration (Figures 3A–E; red point). BVs from the normal skin, found in both the papillary and reticular dermis, expressed CD31 on endothelial cells, which formed a continuous and uniform monolayer (Figure 3F). Arterioles presented the CD31-positive immunoreaction in the endothelial cells in contact with the lumen of the vessel (Figure 3, G). In painless neuropathy, more irregular shaped arterioles were seen (Figure 3L, M); CD31-positive endothelial cells in contact with the lumen of BVs displayed some irregular projections (Figure 3L); furthermore, some regions of the lining cells were close together and linked by junctions, while others were separated from each other (arrow in Figure 3L). In pain-DDSP, CD31-positive cells in arterioles can be observed in the monolayer or multiple layers; cells formed two, three, or perhaps more incomplete layers, and cell projections extended between layers (Figures 3N, O); moreover, the lining cells exhibited bizarre branching shapes with irregular projections into the lumen (arrow in Figure 3O). In the papillary dermis of patients with neuropathy, a reduced capillary luminal area was observed (Figures 3H, I, K; red arrow).

### 3.3 Immunostaining of ion channels PIEZO1 and PIEZO2 in cutaneous blood vessels from subjects with painless and painful neuropathy

**FIGURE 4:**
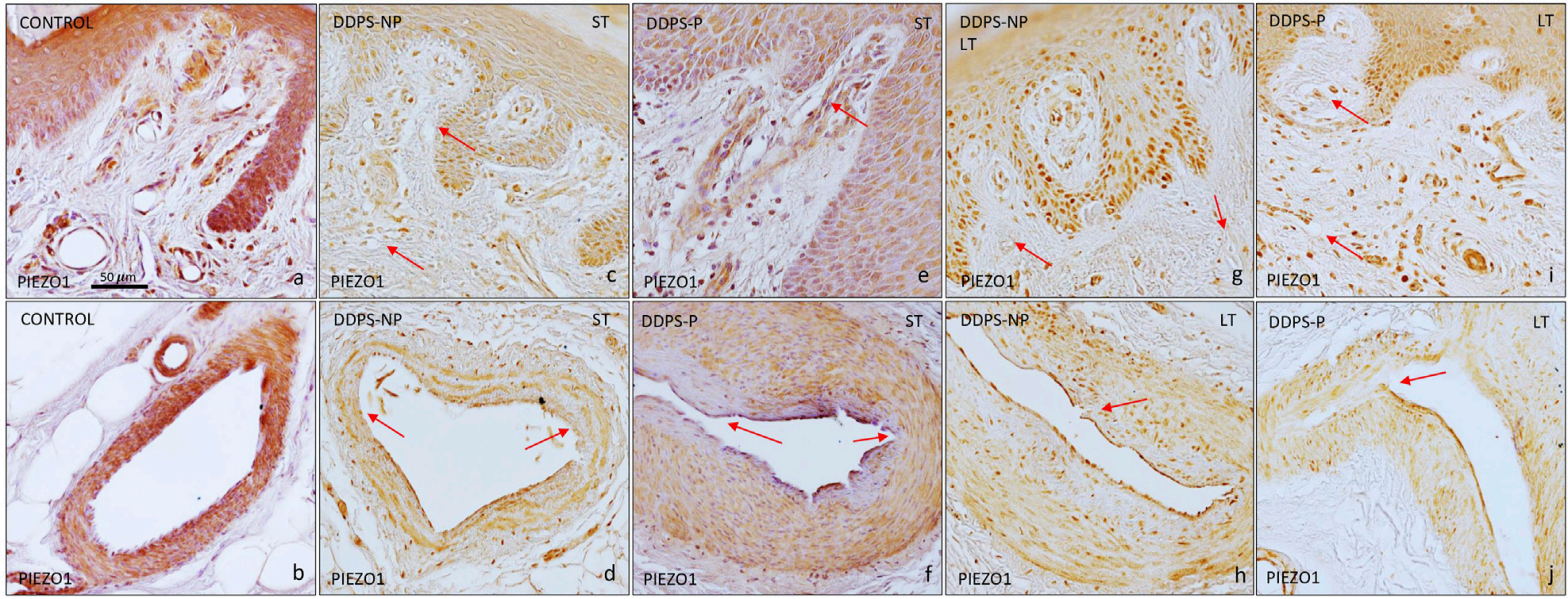
Expression of the PIEZO1 ion channel in the glabrous skin of the feet. The immunoreaction in healthy controls is intense in the endothelium of the vessels of the papillary dermis **(A)** and in the endothelium and muscle layer of the arterioles **(B)**. In patients with neuropathy, there is almost no immunoreaction in the endothelium of papillary vessels [red arrows indicate PIEZO1-negative vessels in **(C, E, G, I)]**. The muscular layer of the arteries presents diffuse labeling in diabetic patients, as well as a discontinuous endothelium [red arrows indicate loss of endothelial continuity in **(D, F, H, J)**].

The immunoreaction was intense for the Piezo1 ion channel in vessels located in both the papillary and reticular dermis of healthy individuals (Figures 4A, B). The immunolabeling was found in the endothelium in contact with the lumen of the vessel (Figure 4A) and in arterioles, where it was also located in the smooth muscle cells (Figure 4B). Patients with DDSP exhibited a decrease in the immunostaining intensity in BVs, which was very evident in arterioles in both the endothelial and muscle cells from all study groups (Figures 4D, F, H, J). In the reticular and papillary dermis, the immunoreaction was slight and there were even BVs without immunolabeling (red arrows in Figures 4C, E, G, I).

**FIGURE 5:**
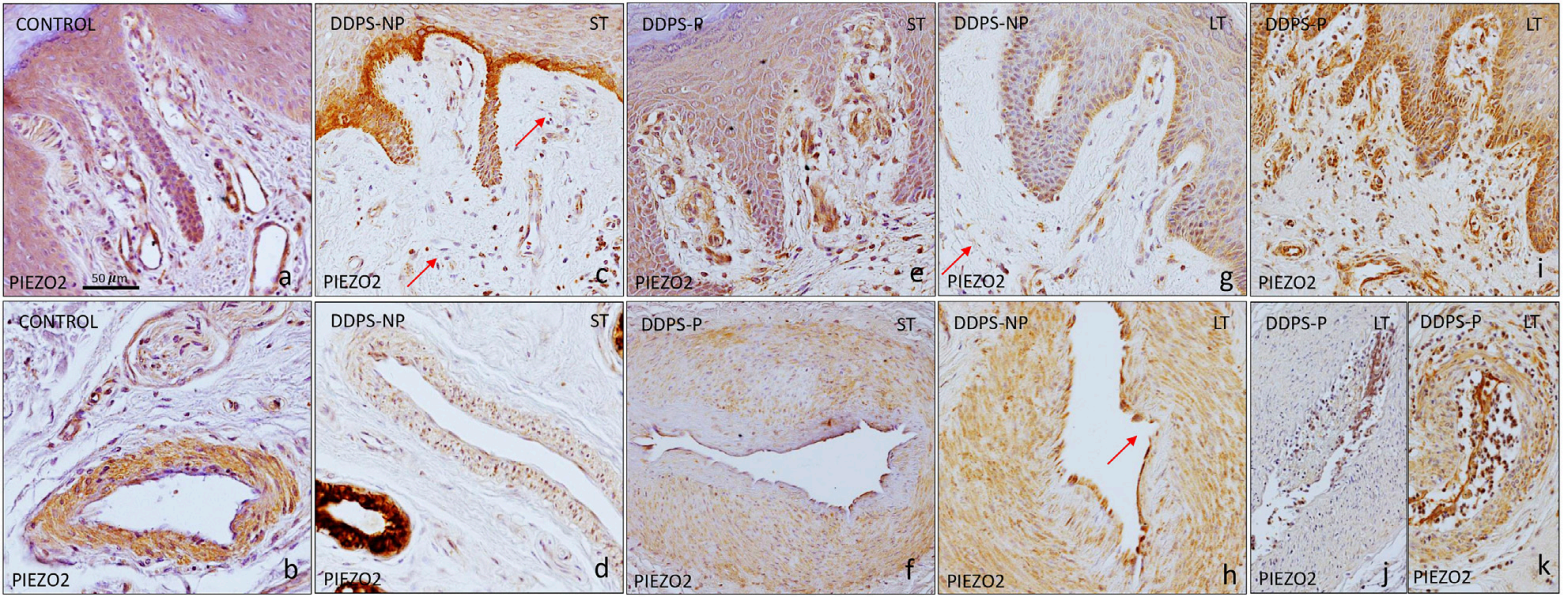
Immunoexpression of PIEZO2 in the vessels of the glabrous skin of the feet. In the healthy controls, an immunoreaction is observed in the endothelium of the veins **(A)**, as well as in the muscular layer of the arteries **(B)**. In patients with DDPS-NP, **(C,D,G,H)** the intensity of the reaction is lower and several blood vessel are negative [red arrows in **(C,G)**]; Moreover ie cells clot to endothelium are arectuve ub arterues [red arrow in **(H)**] . Patients with DDPS-P present a more intense immunoreaction in the papillary vessel **(E,I)**, while arteries in the reticular dermis show a diffuse immunoreaction with respect to healthy individuals **(F,K,J)**.

With respect to Piezo2, the skin of healthy individuals displayed a strong immunoreaction localized in endothelial cells and α-SMA in both venules and arterioles (Figures 5A, B). Individuals with short- and long-term painless neuropathy in the papillary dermis showed weak immunoreaction and even non-immunoreactive vessels (red arrow in Figures 5C, G). Regarding arterioles, a decrease in the immunoreaction intensity in both the endothelium and smooth muscle fibers was also shown (Figures 5D, H); patients with LT-DDSP presented immunoreaction for Piezo2 in the endothelium without continuity in arterioles and lack of expression in pericytes or smooth muscular cells adjacent to the endothelium (arrow in Figure 5H). Patients with painful neuropathy had intense immunostaining for this channel in papillary dermis vessels, which was more intense in case of longstanding diabetes; negative vessels were not seen (Figures 5E, I); however, arterioles presented an immunoreaction similar to the group with painless DPPN because of immunostaining decrease in the endothelium and adjacent smooth muscle cells (Figures 5F, J, K).

**FIGURE 6:**
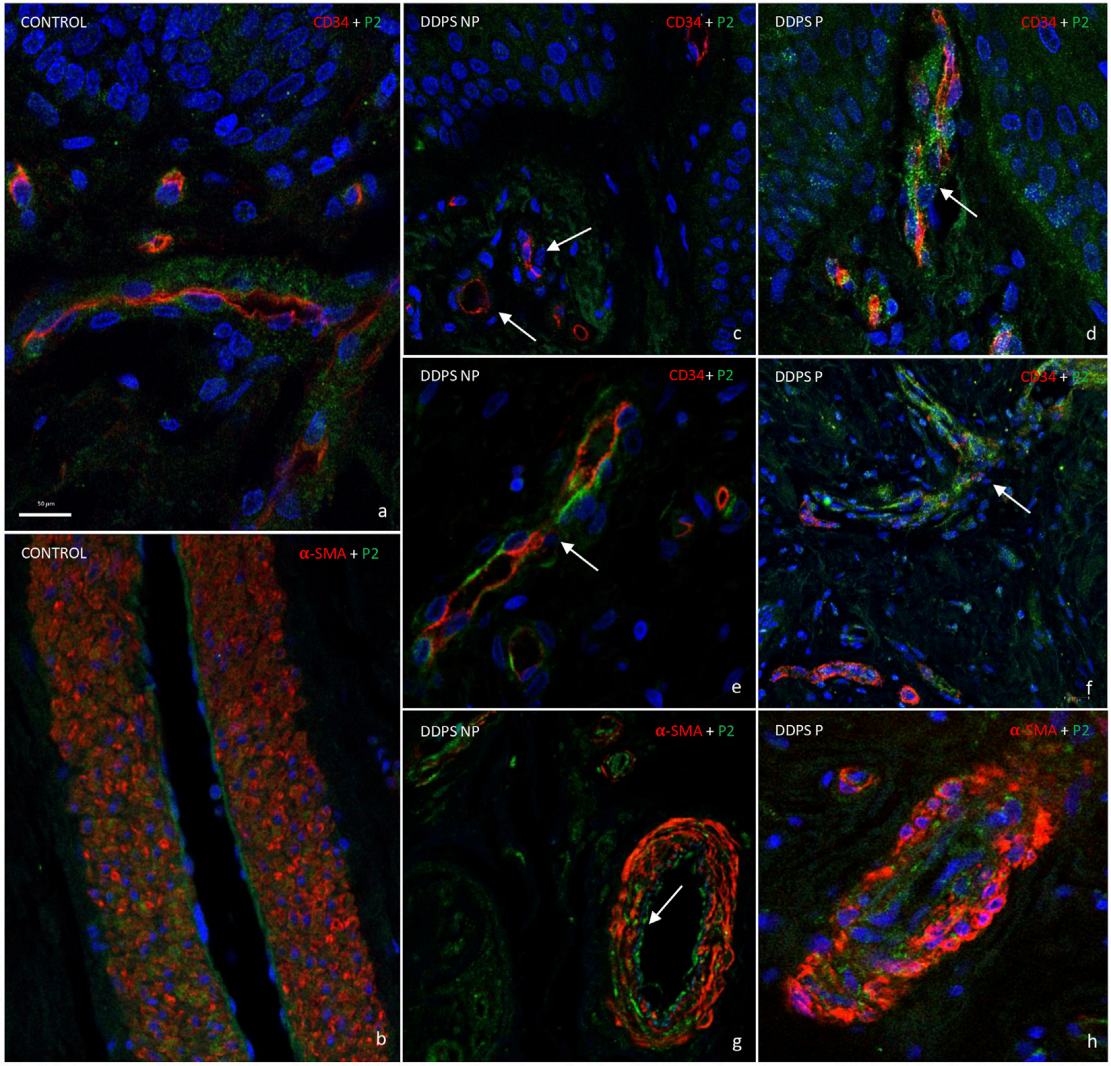
Double fluorescence of CD34/α-SMA and PIEZO2 ion channels in blood vessels. Healthy individuals show PIEZO2 expression in the endothelium of blood vessels **(A)** and arteries **(B)**. Patients with painless diabetic neuropathy show a loss of immunoreaction [white arrow in **(C, E, G)**]. However, PIEZO2 immunostaining in green is higher in patients with painful neuropathy **(D, F, H)**, being more striking in the endothelium of the vessels of the papillary dermis [see arrow in **(D, F)**]. Objective ×63/1.40 oil; pinhole 1.37; xy resolution 139.4 nm and Z resolution 235.8 nm.

To confirm that Piezo2 occurrence in BVs in DDSP patients may be related with pain, double immunofluorescence for BV markers (CD34 and α-SMA) and Piezo2 was performed by confocal microscopy (Figure 6).

In agreement with the data described previously, Piezo2 immunostaining was apparently localized in the endothelium and muscular layer in healthy individuals (Figures 6A, B). Piezo2 immunostaining (green) was placed around CD34-positive cells in endothelial cells (red) (Figure Fig6A) and in flattened cells inside α-SMA cells (red; Figure 6B) in healthy subjects. The immunofluorescence of Piezo2 channels in BVs could be determined by topographical localization (Figures 6A, B), although it is also obvious that there was not apparent co-localization of both proteins (merged images in yellow; not seen). This discrepancy could explain because both proteins, CD34 and α-SMA, were expressed in high intensity, which masked the Piezo2 immunofluorescence; moreover, CD34 immunoreaction exclusively showed an intense linear labeling at the luminal aspect of the BV endothelium, whereas Piezo2 staining (in green) was diffuse, including the cytoplasm of endothelial cells (Figures 6A, C–F).

Therefore, we applied the same double immunofluorescence in painless and painful LT-DDSP skin. In the papillary dermis, the images obtained showed a weak or lack of immunoreaction for Piezo 2 (green) in CD34-positive (red) BVs (see the arrow in Figure 6C) of painless patients, while an intense immunostaining (green) was observed around CD34-positive (red) BVs for painful DDSP patients (see the arrow in Figure 6D). In the reticular dermis, we observed the same results as in the papillary dermis in relation with Piezo2 staining with an intense immunoreaction in DDSP-P (Figure 6E, F). Moreover, CD34 immunofluorescence (red) was discontinuous in some endothelium as we described previously on simple immunohistochemistry (white arrow in Figure 6E). Finally, α-SMA immunofluorescence (red) showed that smooth muscle cells presented on DDPN arterioles lost the packing (Figures 6G, H). Piezo2 is localized in the endothelium in a discontinuous manner in some cases (white arrow in Figure 6G) and between BV muscle cells (Figures 6G, H).

## 4 Discussion

The aim of this research was to try to identify the differences between painful and painless DPN, looking for phenotypes in toes skin BV morphology and distribution associated with pain.

The literature about patients with neuropathy has reported alterations of capillary circulation in the skin from patients with DDPN (Netten et al., 1996; Quattrini et al., 2007; Wang et al., 2022) and an increase in the number of BVs (Adamska et al., 2019; Shillo et al., 2021). In the present study, we used the most common vascular markers for the evaluation of BV density, i.e., CD34 and CD31 (Pusztaszeri et al., 2006), and found an increase in the MVD in the DDPN skin as was previously reported (Adamska et al., 2019; Shillo et al., 2021). The median MVD defined by CD34 protein expression was increased when compared with the number of CD31-positive BV in ST-DDSP, the opposite of the work of Adamska et al. (2019). This discrepancy may be due to differences between studies, type I *versus* II diabetes, glabrous *versus* hairy skin, or young people (over 40) *versus* old people (more than 63 years). In the LT-DDSP group, the results were different; we found a higher increase for CD31^+^ BV with respect to healthy control, whereas MVD for CD34 was the same as for healthy individuals. In light of these results, it seems that the CD31 antigen may be the most promising marker to confirm the association between BV increase and diabetic neuropathy at short evolution (less than 15 years) and the CD34 antigen at long evolution.

Recently, punch skin biopsy studies in type II diabetes indicated that skin microcirculation may be involved in the pathogenesis of painful DPN; in fact, Shillo et al. (2021) demonstrated a significant increase in MVB in subjects with DDPN, particularly in the painful DDPN group using dermal von Willebrand factor (vWF) as a BV marker. This study underlies that the painful DDSP group always has a greater number of BV. Thus, CD34 immunodetection indicates an increase in BV in the painful group with respect to the painless group in ST-DPN; however, the LT DDPN group has more CD31-positive BVs in the painful DDPN group.

Moreover, our research carried out a semiquantitative analysis in both layers of the dermis in all groups. We found an increase in immunopositive BVs in both dermis layers in DDPN patients for both vascular markers. These results suggest that there are slight differences in MDV between the dermis layers. Thus, painful MVD with respect to painless DDPN patients showed an increase in CD31- and CD34-positive BVs, mainly in the reticular dermis in long-term and short-term diabetes, respectively. The results indicate that an increase in BVs is more evident in the reticular dermis.

Overall, the present study confirmed previous studies that established an association between the increase of vessels, DDPN, and pain (Adamska et al., 2019; Shillo et al., 2021). Moreover, the obtained results point out that the most promising method to evaluate painful and painless neuropathic BVs in ST-DDSP is the CD34 marker, whereas the CD31 antibody is adequate for LT-DDSP.

Several classic research studies have reported abnormalities in this patients with mild-to-severe DPN in relation to the BV morphological changes induced by DPN, which include basement membrane thickening, pericyte degeneration, and endothelial cell hyperplasia and hypertrophy (Yasuda and Dyck, 1987; Malik et al., 1989; Malik and Fenton, 1992). To our knowledge, there are no published data about the immunostaining profile with specific markers for skin BVs (CD31, CD34, and α-MCA) of healthy subjects compared to patients with DDSP-P and DDSP-NP, considering the time of disease evolution. A monoclonal antibody that consistently and specifically reacts with BVs permits us to observe a microangiopathy associated with DPN as demonstrated by previous studies (Adamska et al., 2019; Shillo et al., 2019; Shillo et al., 2021). We found some BVs completely or partially collapsed by material. Most of the BVs had a patent lumen delineated by a tortuous and irregular disarrangement in the vascular wall, even leading to its rupture in the affected toe in DPN compared to controls, as previously observed (Malik and Fenton, 1992; Ward, 1993). Moreover, we must note that these results, when comparing DDSP-P and DDSP-NP, indicate that vascular impairment is greater in painful than that in painless DDSP, so this disorganization of the vascular wall could be related to peripheral pain, since it has been reported that the vascular endothelium plays a role in pain mechanisms (Joseph et al., 2013; Joseph et al., 2014). On the other hand, these results are in good agreement with our previous studies about human cutaneous sensory corpuscles in DDSP patients where it was demonstrated that corpuscular alterations were greater in painful DDSP than those in painless DDSP (García-Mesa et al., 2021).

A crucial link in the microvascular etiology of DPN is endothelial dysfunction and inappropriate local blood flow regulation (for a review, see the work of Bönhof et al. (2019)); thus, previous studies have demonstrated that the presence of DPN is accompanied by the impairment of cutaneous endothelium-related vasodilatation (Hamdy et al., 2001; Quattrini et al., 2007) and lower capillary blood flow (Arora et al., 1998; Nabuurs-Fransen et al., 2002; Zhou et al., 2022) in the foot skin. The literature has demonstrated that Piezo mechanosensitive ion channels are expressed in BVs and are involved in the integrated response of BVs to changes in pressure, vascular permeability, and vascular integrity (for a review, see the work of Wu et al. (2017) and Fang et al. (2021)). However, until now, no one has studied the distribution of Piezo channels in relation to vascular alterations caused by diabetic neuropathy.

In last 10 years, numerous research groups have demonstrated that the Piezo1 channel is a sensor for shear stress in the vasculature. The loss of Piezo1 affects the ability of endothelial cells, altering their alignment to the direction of the flow, (Ranade et al., 2014; Cahalan et al., 2015; Rode et al., 2017; Arishe et al., 2020), and mice with induced endothelium-specific Piezo1 deficiency lost the ability to induce NO (oxide nitric) formation in the endothelium and vasodilatation in response to flow (Li et al., 2014; Wang et al., 2016). Moreover, several research studies have suggested that the Piezo1 channel is involved in regulating vascular tone under pathological conditions (for a review, see the work of Fang et al. (2021)). This study using immunohistochemistry with the Piezo1 marker in skin DPN BVs demonstrates a slight staining compared with healthy individuals; in fact, numerous BVs are negative for the Piezo1 marker. The present result could partially explain why skin BVs in neuropathy patients lost the ability to induce vasodilatation. Moreover, Yoda-1 (Piezo1-agonist) also induces vasorelaxation by mimicking the effect of shear stress on endothelial cells (Wang et al., 2016). These findings may facilitate clinical trials to extend the application of Yoda-1 in patients with DPN and also might shed a new light into clinical treatment for diabetic complications.

In the current experiment, we tested the hypothesis that Piezo2 could be the mechano-transducer in the BVs that mediates pain in painful DPN, since the murine model of cancer chemotherapy-induced painful peripheral neuropathy plays a role in vascular pain for Piezo2 (Ferrari et al., 2015). In support of this hypothesis, we found a strong immunostaining for Piezo2 on vascular smooth muscle and endothelial cells in painful DPN when compared to painless DPN, which was mainly associated with the papillary dermis. It should be noted that in the reticular dermis, arterioles are negative for these ion channels in some cases. Vascular wall impairment may produce the loss of characteristic markers on the surface of the endothelium. In fact, we found BVs with the CD31-negative endothelium (data not shown). In light of these results, the Piezo2 antagonist could be effective in the treatment of endothelial cell-dependent vascular neuropathic pain.

Various limitations to the present study should be considered. These data obtained by semiquantitative analysis are limited by the small sample size, although they show the tendency of CD31-positive higher values in subjects with DDSP as compared with healthy controls, like previous studies developed in DPN patients with other specific BV antibodies (Shillo et al., 2021). However, taking into consideration the difficulty of recruiting participants into the study, the current data would still be of value in terms of providing direct pathological evidence of differences between BV morphology and density, comparing participants with painless and painful DPN in the short- and long-term cases. The current findings might, therefore, be validated in future studies with a larger number of samples. Moreover, additional antibody characterization data should be obtained using comparative knockout data, since antibodies Piezo1 and Piezo2 used in this study are new commercial antibodies and do not yet have bibliographic support.

In conclusion, the results of the present study further expand the association between vascular impairment and neuropathy and reveal an association between peripheral diabetic neuropathy and Piezo mechanosensitive ion channels. Therefore, we hypothesize that the presence of Piezo2 could be a risk factor for the development of pain and deficiency of Piezo1 could be related to a vasodilatation deficiency. The current protocols allowed us to detect cutaneous DPN microangiopathy associated with the presence of CD31 and CD34, pain, and duration of diseases in patients with DDPN. Finally, the expression of the Piezo2 channel in BVs of patients with painful DPN might represent early features preceding the onset of pain in these patients. Further investigation is warranted to determine whether the endothelium immunopositivity for Piezo2 in skin microvasculature offers novel therapeutic targets for the prevention of pain appearance.

## Data Availability

The original contributions presented in the study are included in the article/Supplementary Material; further inquiries can be directed to the corresponding author.

## References

[B1] Adamska A. Araszkiewicz A. Pilacinski S. Gandecka A. Grzekla A. Kowalska K. (2019). Dermal microvessel density and maturity is closely associated with atherogenic dyslipidemia and accumulation of advanced glycation end products in adult patients with type 1 diabetes. Microvasc. Res. 121, 46–51. 10.1016/j.mvr.2018.10.002 30312628

[B2] Archer A. G. Roberts V. C. Watkins P. J. (1984). Blood flow patterns in painful diabetic neuropathy. Diabet 27, 563–567. 10.1007/BF00276968 6530051

[B3] Arishe O. O. Ebeigbe A. B. Webb R. C. (2020). Mechanotransduction and uterine blood flow in preeclampsia: the role of mechanosensing Piezo 1 ion channels. Am. J. Hypertens. 33, 1–9. 10.1093/ajh/hpz158 31545339 PMC7768673

[B4] Arora S. Pomposelli F. LoGerfo F. W. Veves A. (2002). Cutaneous microcirculation in the neuropathic diabetic foot improves significantly but not completely after successful lower extremity revascularization. J. Vasc. Surg. 35, 501–505. 10.1067/mva.2002.121126 11877698

[B5] Arora S. Smakowski P. Frykberg R. G. Simeone L. R. Freeman R. LoGerfo F. W. (1998). Differences in foot and forearm skin microcirculation in diabetic patients with and without neuropathy. Diabet. Car. 21, 1339–1344. 10.2337/diacare.21.8.1339 9702444

[B6] Beech D. J. Kalli A. C. (2019). Force sensing by Piezo channels in cardiovascular health and disease. Arterioscler. Thromb. Vasc. Biol. 39, 2228–2239. 10.1161/ATVBAHA.119.313348 31533470 PMC6818984

[B7] Bierhaus A. Nawroth P. P. (2004). Antiangiogenic properties of low molecular weight heparin - does tissue factor provide the answer? Thromb. Haemost. 92, 438–439. 10.1160/TH04-07-0421 15351837

[B8] Bönhof G. J. Herder C. Strom A. Papanas N. Roden M. Ziegler D. (2019). Emerging biomarkers, tools, and treatments for diabetic polyneuropathy. Endocr. Rev. 40, 153–192. 10.1210/er.2018-00107 30256929

[B9] Boulton A. J. Vileikyte L. Ragnarson-Tennvall G. Apelqvist J. (2005). The global burden of diabetic foot disease. Lancet 366, 1719–1724. 10.1016/S0140-6736(05)67698-2 16291066

[B10] Cahalan S. M. Lukacs V. Ranade S. S. Chien S. Bandell M. Patapoutian A. (2015). Piezo1 links mechanical forces to red blood cell volume. eLife 4, e07370. 10.7554/eLife.07370 26001274 PMC4456639

[B11] DeLisser H. M. Christofidou-Solomidou M. Strieter R. M. Burdick M. D. Robinson C. S. Wexler R. S. (1997). Involvement of endothelial PECAM-1/CD31 in angiogenesis. A. J. Path. 151, 671–677. PMC1857836 9284815

[B12] Doupis J. Grigoropoulou P. Voulgari C. Stylianou A. Georga A. Thomakos P. (2008). High rates of comorbid conditions in patients with type 2 diabetes and foot ulcers. Wounds 20, 132–138. 25942413

[B13] Fang X. Z. Zhou T. Xu J. Q. Wang Y. X. Sun M. M. He Y. J. (2021). Structure, kinetic properties and biological function of mechanosensitive Piezo channels. Cell. Biosci. 11, 13. 10.1186/s13578-020-00522-z 33422128 PMC7796548

[B14] Ferrari L. F. Bogen O. Green P. Levine J. D. (2015). Contribution of Piezo2 to endothelium-dependent pain. Mol. Pain. 11, 65. 10.1186/s12990-015-0068-4 26497944 PMC4619430

[B15] Friedrich E. E. Hong Z. Xiong S. Zhong M. Di A. Rehman J. (2019). Endothelial cell Piezo1 mediates pressure-induced lung vascular hyperpermeability via disruption of adherens junctions. Proc. Natl. Acad. Sci. USA. 2116, 12980–12985. 10.1073/pnas.1902165116 PMC6600969 31186359

[B16] García-Mesa Y. Feito J. González-Gay M. Martínez I. García-Piqueras J. Martín-Cruces J. (2021). Involvement of cutaneous sensory corpuscles in non-painful and painful diabetic neuropathy. J. Clin. Med. 10 (19), 4609. 10.3390/jcm10194609 34640627 PMC8509589

[B17] Ge J. Li W. Zhao Q. Li N. Chen M. Zhi P. (2015). Architecture of the mammalian mechanosensitive Piezo1 channel. Nature 527, 64–69. 10.1038/nature15247 26390154

[B18] Hamdy S. Aziz Q. Thompson D. G. Rothwell J. C. (2001). Physiology and pathophysiology of the swallowing area of human motor cortex. Neural. Plast. 8, 91–97. 10.1155/NP.2001.91 11530891 PMC2565392

[B19] Joseph E. K. Green P. G. Bogen O. Alvarez P. Levine J. D. (2013). Vascular endothelial cells mediate mechanical stimulation-induced enhancement of endothelin hyperalgesia via activation of P2X2/3 receptors on nociceptors. J. Neurosci. 33, 2849–2859. 10.1523/JNEUROSCI.3229-12.2013 23407944 PMC3711399

[B20] Joseph E. K. Green P. G. Levine J. D. (2014). ATP release mechanisms of endothelial cell-mediated stimulus-dependent hyperalgesia. J. Pain. 15, 771–777. 10.1016/j.jpain.2014.04.005 24793242 PMC4264525

[B21] Lai A. Chen Y. C. Cox C. D. Jaworowski A. Peter K. Baratchi S. (2021). Analyzing the shear-induced sensitization of mechanosensitive ion channel Piezo-1 in human aortic endothelial cells. J. Cell. Physiol. 236, 2976–2987. 10.1002/jcp.30056 32959903

[B22] Li J. Hou B. Tumova S. Muraki K. Bruns A. Ludlow M. J. (2014). Piezo1 integration of vascular architecture with physiological force. Nature 515, 279–282. 10.1038/nature13701 25119035 PMC4230887

[B23] Li W. Gao N. Yang M. (2017). The structural basis for sensing by the Piezo1 protein. Curr. Top. Membr. 79, 135–158. 10.1016/bs.ctm.2016.10.001 28728815

[B24] Malik A. B. Fenton J. W. (1992). Thrombin-mediated increase in vascular endothelial permeability. Sem. Throm. Hem. 18, 193–199. 10.1055/s-2007-1002425 1631567

[B25] Malik A. B. Lynch J. J. Cooper J. A. (1989). Endothelial barrier function. J. Invest. Dermatol. 93, 62S-67S–67S. 10.1111/1523-1747.ep12581072 2546995

[B26] Martinac B. Cox C. D. (2017). “Mechanosensory transduction: focus on ion channels,” in Comprehensive biophysics (Elsevier). 10.1016/B978-0-12-809633-8.08094-8

[B27] Matsumura T. Wolff K. Petzelbauer P. (1997). Endothelial cell tube formation depends on cadherin 5 and CD31 interactions with filamentous actin. J. Immunol. 158, 3408–3416. 9120301

[B28] Nabuurs-Franssen M. H. Houben A. J. Tooke J. E. Schaper N. C. (2002). The effect of polyneuropathy on foot microcirculation in Type II diabetes. Diabet 45, 1164–1171. 10.1007/s00125-002-0872-z 12189447

[B29] Netten P. M. Wollersheim H. Thien T. Lutterman J. A. (1996). Skin microcirculation of the foot in diabetic neuropathy. Clin. Sci. (Lond). 91, 559–565. 10.1042/cs0910559 8942394

[B30] Nourse J. L. Pathak M. M. (2017). How cells channel their stress: interplay between Piezo1 and the cytoskeleton. Semin. Cell. Dev. Biol. 71, 3–12. 10.1016/j.semcdb.2017.06.018 28676421 PMC6070642

[B31] Otto M. Bucher C. Liu W. Müller M. Schmidt T. Kardell M. (2020). 12(S)-HETE mediates diabetes-induced endothelial dysfunction by activating intracellular endothelial cell TRPV1. J. Clin. Inves. 130, 4999–5010. 10.1172/JCI136621 PMC7456227 32584793

[B32] Pop-Busui R. Boulton A. J. Feldman E. L. Bril V. Freeman R. Malik R. A. (2017). Diabetic neuropathy: a position statement by the American diabetes association. Diabet. Care. 40, 136–154. 10.2337/dc16-2042 PMC6977405 27999003

[B33] Pusztaszeri M. P. Seelentag W. Bosman F. T. (2006). Immunohistochemical expression of endothelial markers CD31, CD34, von Willebrand factor, and Fli-1 in normal human tissues. J. Histochem. Cytochem. 54, 385–395. 10.1369/jhc.4A6514.2005 16234507

[B34] Quattrini C. Harris N. D. Malik R. A. Tesfaye S. (2007). Impaired skin microvascular reactivity in painful diabetic neuropathy. Diabet. Care. 30, 655–659. 10.2337/dc06-2154 17327336

[B35] Ranade S. S. Qiu Z. Woo S. H. Hur S. S. Murthy S. E. Cahalan S. M. (2014). Piezo1, a mechanically activated ion channel, is required for vascular development in mice. Proc. Natl. Acad. Sci. USA. 111, 10347–10352. 10.1073/pnas.1409233111 24958852 PMC4104881

[B36] Retailleau K. Duprat F. Arhatte M. Ranade S. S. Peyronnet R. Martins J. R. (2015). Piezo1 in smooth muscle cells is involved in hypertension-dependent arterial remodeling. Cell. Rep. 13, 1161–1171. 10.1016/j.celrep.2015.09.072 26526998

[B37] Rode B. Shi J. Endesh N. Drinkhill M. J. Webster P. J. Lotteau S. J. (2017). Piezo1 channels sense whole body physical activity to reset cardiovascular homeostasis and enhance performance. Nat. Commun. 8, 350. 10.1038/s41467-017-00429-3 28839146 PMC5571199

[B38] Schmiedel O. Nurmikko T. J. Schroeter M. L. Whitaker R. Harvey J. N. (2008). Alpha adrenoceptor agonist-induced microcirculatory oscillations are reduced in diabetic neuropathy. Microvasc. Res. 76, 124–131. 10.1016/j.mvr.2008.04.004 18602650

[B39] Shillo P. Sloan G. Greig M. Hunt L. Selvarajah D. Elliott J. (2019). Painful and painless diabetic neuropathies: what is the difference? Curr. Diab. Rep. 19, 32. 10.1007/s11892-019-1150-5 31065863 PMC6505492

[B40] Shillo P. Yiangou Y. Donatien P. Greig M. Selvarajah D. Wilkinson I. D. (2021). Nerve and vascular biomarkers in skin biopsies differentiate painful from painless peripheral neuropathy in type 2 diabetes. Front. Pain. Res. (Lausanne) 2, 731658. 10.3389/fpain.2021.731658 35295465 PMC8915761

[B41] Stirban A. (2014). Microvascular dysfunction in the context of diabetic neuropathy. Curr. Diab. Rep. 14, 541. 10.1007/s11892-014-0541-x 25189434

[B42] Tesfaye S. Boulton A. J. Dyck P. J. Freeman R. Horowitz M. Kempler P. Toronto Diabetic Neuropathy Expert Group (2010). Diabetic neuropathies: update on definitions, diagnostic criteria, estimation of severity, and treatments. Diabet. Care. 33, 2285–2293. 10.2337/dc10-1303 PMC2945176 20876709

[B43] Tomešová J. Gruberova J. Lacigova S. Cechurova D. Jankovec Z. Rusavy Z. (2013). Differences in skin microcirculation on the upper and lower extremities in patients with diabetes mellitus: relationship of diabetic neuropathy and skin microcirculation. Diabet. Technol. Ther. 15, 968–975. 10.1089/dia.2013.0083 23964895

[B44] Wang G. Lu P. Qiao P. Zhang P. Cai X. Tang L. (2022). Blood vessel remodeling in late stage of vascular network reconstruction is essential for peripheral nerve regeneration. Bioeng. Transl. Med. 7, e10361. 10.1002/btm2.10361 36176610 PMC9472024

[B45] Wang S. Chennupati R. Kaur H. Iring A. Wettschureck N. Offermanns S. (2016). Endothelial cation channel PIEZO1 controls blood pressure by mediating flow-induced ATP release. J. Clin. Invest. 126, 4527–4536. 10.1172/JCI87343 27797339 PMC5127677

[B46] Ward J. D. (1993). Abnormal microvasculature in diabetic neuropathy. Eye (Lond). 7, 223–226. 10.1038/eye.1993.53 7607339

[B47] Wu J. Lewis A. H. Grandl J. (2017). Touch, tension, and transduction - the function and regulation of Piezo ion channels. Trends. biochem. Sci. 42, 57–71. 10.1016/j.tibs.2016.09.004 27743844 PMC5407468

[B48] Yang H. Liu C. Zhou R. M. Yao J. Li X. M. Shen Y. (2016). Piezo2 protein: a novel regulator of tumor angiogenesis and hyperpermeability. Oncotarget 7, 44630–44643. 10.18632/oncotarget.10134 27329839 PMC5190124

[B49] Yao X. Qian C. N. Zhang Z. F. Tan M. H. Kort E. J. Yang X. J. (2007). Two distinct types of blood vessels in clear cell renal cell carcinoma have contrasting prognostic implications. Clin. Cancer Res. 13 (1), 161–169. 10.1158/1078-0432.CCR-06-0774 17200351

[B50] Yasuda H. Dyck P. J. (1987). Abnormalities of endoneurial microvessels and sural nerve pathology in diabetic neuropathy. Neurol 37, 20–28. 10.1212/wnl.37.1.20 3796834

[B51] Zhou Q. Qian Z. Yang M. Liu J. Wu J. Ren L. (2022). Alterations in plantar vessel blood flow in patients with mild diabetic peripheral neuropathy. BMJ. Open. Diabet. Res. Care. 10, e002492. 10.1136/bmjdrc-2021-002492 PMC8762148 35027366

