# Supplementary material for: Relationship of PIEZO1 and PIEZO2 vascular expression with diabetic neuropathy
Source: Front Physiol. 2023 Nov 20;14:1243966. doi: 10.3389/fphys.2023.1243966 (PMC10694834; doi:10.3389/fphys.2023.1243966)
Supplement: Supplementary file 1 [file Table1.DOCX]

| Antigen | Origin | Dilution | Epitope | Homology | Supplier | Stock |
| --- | --- | --- | --- | --- | --- | --- |
| PIEZO1 (LS-B156/12883) | Rabbit | 1:200 | UniProt Q92508 | See: <https://www.uniprot.org/uniref/UniRef90_Q92508> | Lifespan Biosciences, Seattle, WA, USA | In stock |
| PIEZO2 (63895) | Rabbit | 1:500 | UniProtKB Q9H5I5 | See: <https://www.orthodb.org/?ncbi=63895> | Sigma-Aldrich, Madrid, Spain | In stock |
|  |  |  |  |  |  |  |
