# Supplementary figures and images for: Relationship of PIEZO1 and PIEZO2 vascular expression with diabetic neuropathy
Source: Front Physiol. 2023 Nov 20;14:1243966. doi: 10.3389/fphys.2023.1243966 (PMC10694834; doi:10.3389/fphys.2023.1243966)

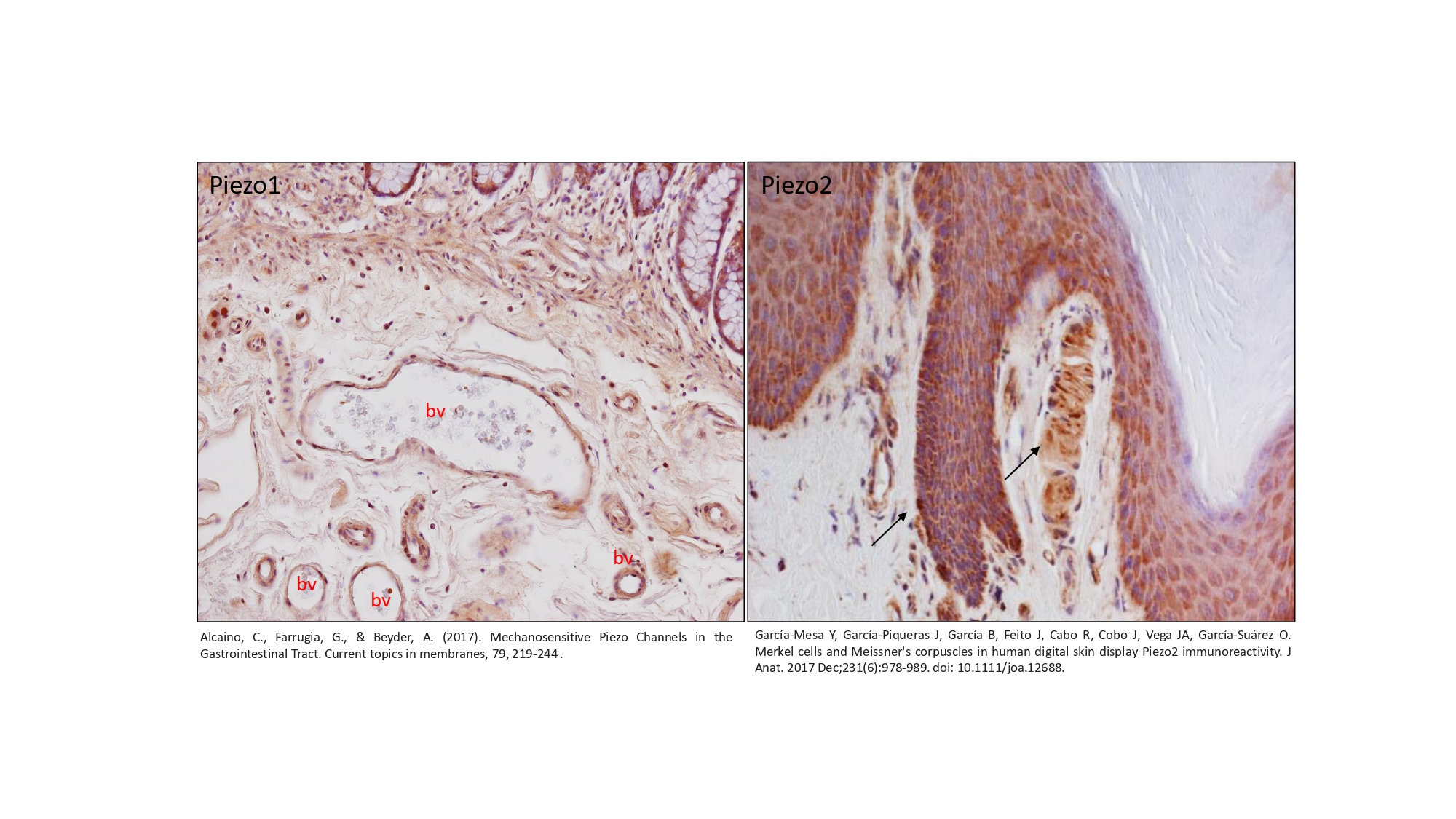

Supplement: Supplementary file 2 [file Image1.JPEG]
